# A novel aGAPSS-based nomogram for the prediction of ischemic stroke in patients with antiphospholipid syndrome

**DOI:** 10.3389/fimmu.2022.930087

**Published:** 2022-07-29

**Authors:** Xiaodong Song, Yangyi Fan, Yuan Jia, Gongming Li, Meige Liu, Yicheng Xu, Jun Zhang, Chun Li

**Affiliations:** ^1^ Department of Neurology, Peking University People’s Hospital, Beijing, China; ^2^ Department of Rheumatology and Immunology, Peking University People’s Hospital, Beijing, China; ^3^ Department of Rheumatology and Immunology, Linyi Traditional Chinese Medicine Hospital, Linyi, China; ^4^ Department of Neurology, Aerospace Center Hospital, Beijing, China

**Keywords:** antiphospholipid syndrome, adjusted Global Anti-Phospholipid Syndrome Score, ischemic stroke, nomogram, risk stratification

## Abstract

**Background:**

Ischemic stroke (IS) is the most common and life-threatening arterial manifestation of antiphospholipid syndrome (APS). It is related to high mortality and severe permanent disability in survivors. Thus, it is essential to identify patients with APS at high risk of IS and adopt individual-level preventive measures. This study was conducted to identify risk factors for IS in patients with APS and to develop a nomogram specifically for IS prediction in these patients by combining the adjusted Global Anti-Phospholipid Syndrome Score (aGAPSS) with additional clinical and laboratory data.

**Methods:**

A total of 478 consecutive patients with APS were enrolled retrospectively. All patients were randomly assigned to the training and validation cohorts. Univariate and multivariate binary logistic analyses were conducted to identify predictors of IS in the training cohort. Then, a nomogram was developed based on these predictors. The predictive performance of the nomogram for the training and validation cohorts was evaluated by determining areas under the receiver operating characteristic curve (AUROC) and creating calibration plots. A decision curve analysis (DCA) was conducted to compare the potential net benefits of the nomogram with those of the aGAPSS.

**Results:**

During a mean follow-up period of 2.7 years, 26.9% (129/478) of the patients were diagnosed with IS. Binary logistic regression analysis revealed that five risk factors were independent clinical predictors of IS: age (*P* < 0.001), diabetes (*P* = 0.030), hyperuricemia (*P* < 0.001), the platelet count (*P* = 0.001), and the aGAPSS (*P* = 0.001). These predictors were incorporated into the nomogram, named the aGAPSS-IS. The nomogram showed satisfactory performance in the training [AUROC = 0.853 (95% CI, 0.802–0.896] and validation [AUROC = 0.793 (95% CI, 0.737–0.843)] cohorts. Calibration curves showed good concordance between observed and nomogram-predicted probability in the training and validation cohorts. The DCA confirmed that the aGAPSS-IS provided more net benefits than the aGAPSS in both cohorts.

**Conclusion:**

Age, diabetes, hyperuricemia, the platelet count, and the aGAPSS were risk factors for IS in patients with APS. The aGAPSS-IS may be a good tool for IS risk stratification for patients with APS based on routinely available data.

## Introduction

Antiphospholipid syndrome (APS) is an autoimmune disorder characterized by recurrent thrombotic events and pregnancy morbidity associated with the persistent presence of antiphospholipid antibodies (aPLs) (1). Ischemic stroke (IS) is one of the most common central nervous system manifestations and the most life-threatening complication of APS (2, 3). It accounts for nearly half of the arterial events caused by APS (4). Nearly 20% of cerebral strokes in patients younger than 50 years have been suggested to be associated with APS (5). In addition, cerebral infarction was reported in 19.8% of a cohort of 1000 European patients with APS and accounted for 11.8% of all deaths that occurred during a 10-years follow-up period (6). IS is associated with a high mortality rate and severe permanent disability in survivors (7). In 2019, stroke led to 6.55 million deaths and 143 million disability-adjusted life years on average, 62.4% of which were ischemic strokes (8). Thus, it is essential to identify patients with APS at high risk of IS and adopt individual-level preventive measures.

No widely accepted predictive tool or model has been established for aPL-positive patients. The Global Antiphospholipid Syndrome Score (GAPSS) and antiphospholipid score (aPL-S) are used to predict thrombosis in patients with APS (9, 10). Although their utility has been validated with various external cohorts, they rely on aPL quantification (11–13), which is difficult due to the problems with the standardization of aPL criteria and the difficulty of interpreting lupus anticoagulant results (14). Furthermore, these scoring systems require data on laboratory parameters not routinely measured in daily clinical practice. GAPSS modifications, including the adjusted Global Antiphospholipid Syndrome Score (aGAPSS) and aGAPSS specific for cardiovascular disease (aGAPSS_CVD_), have been proposed (15, 16), and patients with primary APS who had experienced IS were found to have higher aGAPSS (17). However, little is known about the efficiency of the aGAPSS for the prediction of IS in patients with APS. Traditional thrombotic risk factors, including obesity, smoking habit, and diabetes, also increase the risk of thrombosis in these patients (18).

In the present study, we evaluated the risk factors for IS in patients with APS. We also developed a new nomogram specifically for IS prediction in these patients by combining the aGAPSS with additional risk factors.

## Methods

### Patients and baseline data collection

Consecutive patients with APS who attended Peking University People’s Hospital between 1 January 2005 and 1 March 2021 were enrolled retrospectively in this study. All participants met the 2006 Sydney classification criteria for APS (1). The exclusion criteria were: 1) IS occurrence before APS onset; 2) other coagulation disorders, such as severe hepatic diseases and malignancy; and 3) incomplete medical records. The following clinical data were collected at the time of APS diagnosis: age, sex, body mass index, time from first APS event, history of autoimmune disease (e.g., systemic lupus erythematosus, Sjögren’s syndrome), vascular thrombosis, pregnancy morbidity, hypertension, hyperlipidemia, diabetes, chronic obstructive pulmonary disease (COPD), chronic kidney disease, hyperuricemia, smoking, laboratory data, and treatment. Patients were followed by telephone interviews or clinic visits every three months.

### Assessment of risk factors for ischemic stroke

According to the guidelines of the American Stroke Association (12), hypertension, diabetes, smoking, and hyperlipidemia were considered to be traditional risk factors for IS. Hypertension, diabetes, and smoking were assessed according to the guidelines of the National Institute for Health and Care Excellence (19). Hypertension was defined as high blood pressure at two or more random time points or the use of antihypertensive medication. Diabetes was defined as a fasting blood glucose level > 7.0 mmol/L on more than two occasions or the use of insulin or oral antidiabetic drugs. Smoking status was determined by self-reports of tobacco consumption. According to the Chinese Guideline for the Management of Dyslipidemia in Adults (20), hyperlipidemia was defined by any of the following criteria: 1) triglyceride level > 2.3 mmol/L, 2) high-density lipoprotein level < 1.0 mmol/L, 3) low-density lipoprotein level > 4.1 mmol/L, and 4) total cholesterol level > 6.2 mmol/L. Hyperuricemia was established when fasting serum urate levels equaled to or exceeded 420 μmol/L (21). Thrombocytopenia was defined as a platelet count <100 × 10^9^/L (1). In addition, diagnoses of COPD and chronic kidney disease, recently accepted as risk factors for stroke (22–24), were confirmed by medical record review.

### Antiphospholipid antibodies detection and aGAPSS

Anti-cardiolipin (aCL) and anti-β2-glycoprotein I antibody (aβ2GPI) were detected by enzyme-linked immunosorbent assay as described previously (25). Values for aCL > 12 IU/mL and aβ2GPI > 27 RU/mL were considered positive based on local cut-off. The lupus anticoagulant (LAC) assay was performed using Stago STA Compact Hemostasis System as described previously (25). The simplified Dilute Russell’s Viper Venom Test (dRVVT) was considered positive if the dRVVT ratios were > 1.2.

The aGAPSS was calculated as previously reported by adding corresponding points to the risk factors: 3 for hyperlipidemia, 1 for arterial hypertension, 5 for aCL, 4 for aβ2GPI, and 4 for LAC (10).

### Assessment of ischemic stroke

Two experienced neurologists assigned patients to IS and non-IS groups based on clinical manifestations and neuroimaging (magnetic resonance imaging or computed tomography) findings. The diagnostic criteria for ischemic stroke are as follows: (1) acute onset; (2) focal neurological deficit (weakness or numbness of one side of the face or limb, speech impairment, etc.); (3) presence of a responsible lesion on imaging or signs/symptoms lasting more than 24 h; (4) exclusion of non-vascular causes; and (5) exclusion of cerebral hemorrhage by neuroimaging (26). Any inconsistency in the definition of IS was resolved by a senior neurologist.

### Statistical analysis

The statistical analyses were performed using IBM SPSS Statistics (version 22.0), MedCalc software (version 20.1.0), and R software (version 4.1.2). By using a computer random number generator, one-half of the patients were randomized into the training cohort to construct the predictive nomogram, and the remaining patients were assigned to the validation cohort to evaluate the performance of the nomogram. Group comparisons were performed using the unpaired *t*-test (for normally distributed data) and Mann–Whitney *U* test (for non-normally distributed data) for quantitative variables, and Fisher’s exact test and the chi-squared test for categorical variables. Receiver operating characteristic (ROC) curve analysis was used to determine the cutoff aGAPSS for discrimination of the IS and non-IS groups.

For the training cohort, univariate logistic regression analysis was performed to screen for potential predictors of IS. To identify independent risk factors for IS, variables with *P* values < 0.05 in the univariate analysis were included in a multivariate regression model based on the training cohort. The variance inflation factor (VIF) was used to measure the impact of collinearity among the variables in the regression model. Then, a nomogram was built based on these independent predictors using the rms package of the R software. The area under the receiver operating characteristic curve (AUROC) was drawn to evaluate and compare the discrimination efficacy of the nomogram with that of the aGAPSS. To access the predictive accuracy of the nomogram, calibration curves were drawn by plotting the observed probability against the nomogram-predicted probability. Finally, a decision curve analysis (DCA) was conducted with the rmda package to evaluate and compare potential net benefits at different threshold probabilities. For all statistical tests, two-sided *P* values < 0.05 were significant.

## Results

### Baseline clinical characteristics of study cohort

**Figure 1:**
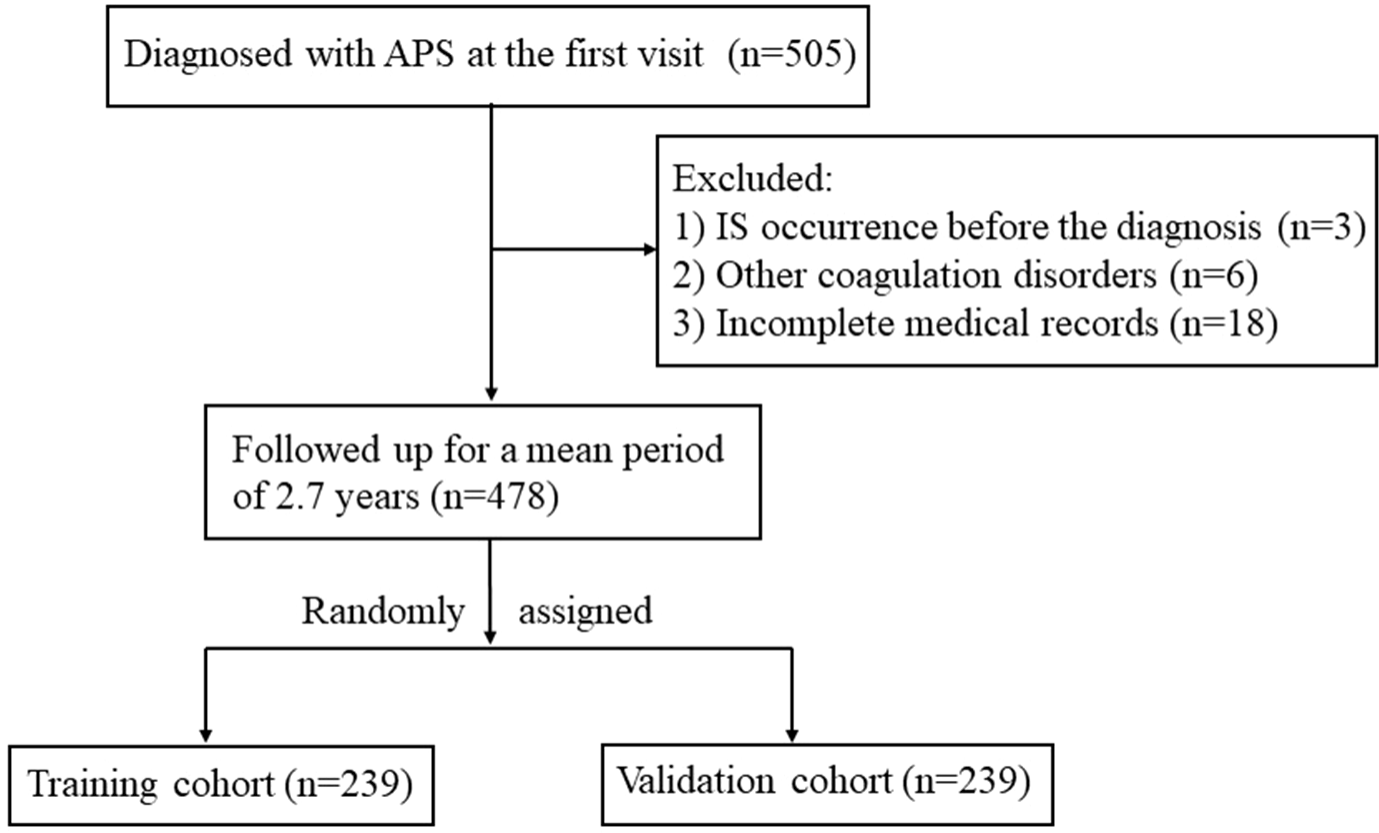
Study flow diagram. IS, ischemic stroke; APS, antiphospholipid antibody syndrome.

Of 505 patients with APS initially identified, three whose IS preceded APS onset, six with other coagulation diseases (four with malignancies and two with severe hepatic disease), and 18 whose medical records were incomplete were excluded. The remaining 478 patients were assigned randomly to the training and validation cohorts (*n* = 239 each; **Figure 1**). Baseline clinical data did not differ between cohorts (**Table 1**). During a mean follow-up period of 2.7 years, 129 (26.9%) patients were diagnosed with IS. Among all the IS patients in our study, eight patients had no definite neurological deficit symptom. Their silent ischemic lesions were identified through MRI scan due to nonspecific symptom (e.g., headache, dizziness), which showed high signal on the diffusion-weighted image (DWI). The DWI sequence has high accuracy for diagnosing IS (88%−100% sensitivity and 95%−100% specificity) (27). The IS incidence rates were similar in the training and validation cohorts [*n* = 69 (28.9%) and *n* = 61 (25.5%), respectively].

**Table 1:** Demographic and clinical variables of APS patients at baseline.

Variable	All cases (n = 478)	Training set (n = 239)	Validation set (n = 239)	P-value
Male, n (%)	112 (23.4)	61 (25.5)	51 (21.3)	0.28
Age (years), median (IQR)	41.0 (31.0-57.0)	42.0 (32.0-57.0)	41 (31-57)	0.94
BMI (kg/m²), median (IQR)	23.6 (20.8-26.4)	23.7 (20.7-26.6)	23.6 (21.0-26.1)	0.71
Time from the first APS event (months), median (IQR)	11.0 (1.0-36.0)	8.0 (1.0-36.0)	8.0 (1.0-36.0)	0.71
aGAPSS, median (IQR)	10.0 (7.0-13.0)	11.0 (7.0-13.0)	10.0 (7.0-14.0)	0.99
Autoimmune disease, n (%)	216 (45.2)	115 (48.1)	101 (42.3)	0.20
Systemic lupus erythematosus, n (%)	152 (31.8)	80 (33.5)	72 (30.1)	0.432
Sjögren’s syndrome, n (%)	34 (7.1)	15 (6.3)	19 (7.9)	0.477
rheumatoid arthritis, n (%)	19 (4.0)	12 (5.0)	7 (2.9)	0.242
systemic sclerosis, n (%)	11 (2.3)	6 (2.5)	5 (2.1)	0.760
Vascular thrombosis only, n (%)	299 (62.6)	156 (65.3)	143 (59.8)	0.22
Pregnancy morbidity only, n (%)	144 (30.1)	70 (29.3)	74 (30.9)	0.69
Vascular thrombosis and pregnancy morbidity, n (%)	35 (7.3)	19 (7.9)	16 (6.7)	0.60
Smoking, n (%)	58 (12.1)	29 (12.1)	29 (12.1)	1.00
Hypertension, n (%)	135 (28.2)	74 (31.0)	61 (25.5)	0.20
Hyperlipidemia, n (%)	248 (51.9)	130 (54.4)	118 (49.4)	0.27
Diabetes, n (%)	66 (13.8)	37 (15.5)	29 (12.1)	0.29
COPD, n (%)	6 (1.3)	4 (1.7)	2 (0.8)	0.69
Chronic kidney disease, n (%)	29 (6.1)	16 (6.7)	13 (5.4)	0.57
Hyperuricemia, n (%)	46 (9.6)	25 (10.5)	21 (8.8)	0.54
Anticoagulation, n (%)	184 (38.5)	95 (39.7)	89 (37.2)	0.57
Antiplatelet, n (%)	133 (27.8)	65 (27.2)	68 (28.5)	0.71
Immunosuppressant, n (%)	203 (42.5)	111 (46.4)	92 (38.5)	0.13
HCQ, n (%)	229 (47.9)	113 (47.3)	116 (48.5)	0.78
aCL, n (%)	299 (62.6)	149 (62.3)	150 (62.8)	0.93
aβ2GPI, n (%)	308 (64.4)	152 (63.6)	156 (65.3)	0.70
LAC, n (%)	281 (58.8)	142 (59.4)	139 (58.2)	0.78
Triple aPL positivity, n (%)	165 (34.5)	75 (31.4)	90 (37.6)	0.15
Platelet (×10^9^/L), median (IQR)	151.0 (76.5-217.0)	153.0 (87.0-225.0)	155.0 (66.1-217.0)	0.31
Mean platelet volume (fl), median (IQR)	9.8 (8.6-10.9)	9.8 (8.6-10.9)	9.8 (8.6-10.9)	0.71
INR, median (IQR)	1.0 (0.9-1.1)	1.0 (0.9-1.2)	1.0 (0.9-1.2)	0.28
D-Dimer (ng/ml), median (IQR),	267.0 (100.0-580.0)	251.0 (94.0-564.0)	222.0 (92.0-544.0)	0.58
ESR increased, n (%)	203 (42.5)	98 (41.0)	105 (43.9)	0.52
CRP increased, n (%)	142 (29.7)	72 (30.1)	70 (29.3)	0.84
Low C3, n (%)	189 (39.5)	99 (41.4)	90 (37.7)	0.40
Low C4, n (%)	185 (38.7)	95 (39.7)	90 (37.7)	0.64
ANA positive, n (%)	279 (58.4)	146 (61.1)	133 (55.6)	0.23

IS, ischemic stroke; APS, antiphospholipid antibody syndrome; BMI, body mass index; IQR, interquartile range; aGAPSS, adjusted Global Anti-Phospholipid Syndrome Score; COPD, chronic obstructive pulmonary disease; HCQ, Hydroxychloroquine; LAC, lupus anticoagulant; aCL, anti-cardiolipin antibody; aβ2GPI, anti-β2-glycoprotein I antibody; aPL, antiphospholipid antibody; IQR, interquartile range; INR, international normalized ratio; C3, complement 3; C4, complement 4; ANA, antinuclear antibody.

### Construction of the predictive nomogram for IS

In the training cohort, patients with IS were older than those without IS [54.0 (43.0–64.0) *vs.* 37.0 (30.0–50.0) years, *P* < 0.01] and the proportion of males was larger in the former group (36.2% *vs.* 21.2%, *P* = 0.02). aGAPSS were higher for patients with than for those without IS [13.0 (11.0–16.0) *vs.* 9.0 (5.3–13.0), *P* < 0.01]. Greater prevalence of hypertension (50.7% *vs.* 24.1%, *P* < 0.01), diabetes (30.4% *vs.* 7.1%, *P* < 0.01), hyperlipidemia (65.2% *vs.* 50%, *P* = 0.03), and hyperuricemia (26.1% *vs.* 4.7%, *P* < 0.01) were also observed in patients with IS than in those without IS. Among laboratory parameters, the platelet count [114.9 (63.0–172.0) *vs.* 180.1 (104.3–237.5) ×10^9^/L, *P* < 0.01] was lower in the IS than in the non-IS group. The rate of aCL positivity (73.9% *vs.* 58.8%, *P* = 0.03), LAC positivity (79.7% *vs.* 53.5%, *P* < 0.01), and triple aPL positivity (44.9% *vs.* 25.9%, P < 0.01), were also higher in the IS than in the non-IS group (**Table 2**).

**Table 2:** Univariate analysis of ischemic stroke occurrence based on the training cohort.

Variable	IS group (n = 69)	non-IS group (n = 170)	OR (95% CI)		P-value
Male, n (%)	25 (36.2)	36 (21.2)	2.115 (1.145-3.906)		**0.017**
Age (years), median (IQR)	54.0 (43.0-64.0)	37.0 (31.0-50.0)	1.055 (1.034-1.076)		**<0.001**
BMI (kg/m²), median (IQR)	24.0 (21.3-27.3)	23.4 (20.7-26.4)	1.021 (0.958-1.087)		0.522
Time from the first APS event (months), median (IQR)	11.0 (2.0-48.0)	7 (1.0-33.2)	1.004 (1.000-1.009)		0.640
aGAPSS, median (IQR)	13.0 (11.0-16.0)	9.0 (5.3-13.0)	1.169 (1.088-1.256)		**<0.001**
Autoimmune disease, n (%)	40 (57.9)	75 (44.1)	1.97 (1.114-3.486)		**0.020**
Smoking, n (%)	12 (17.4)	17 (10.0)	1.895 (0.852-4.213)		0.117
Hypertension, n (%)	35 (50.7)	41 (24.1)	3.239 (1.798-5.834)		<0.001
Hyperlipidemia, n (%)	45 (65.2)	85 (50.0)	1.875 (1.050-3.347)		0.033
Diabetes, n (%)	21 (30.4)	12 (7.1)	8.464 (3.957-18.106)		**<0.001**
COPD, n (%)	3 (4.3)	1 (0.6)	7.682 (0.785-75.176)		0.080
Chronic kidney disease, n (%)	6 (8.7)	10 (5.9)	1.524 (0.531-4.369)		0.433
Hyperuricemia, n (%)	18 (26.1)	8 (4.7)	7.147 (2.934-17.409)		**<0.001**
Anticoagulation, n (%)	24 (34.8)	75 (44.1)	0.676 (0.378-1.207)		0.185
Antiplatelet, n (%)	24 (34.8)	42 (24.7)	1.625 (0.887-2.979)		0.116
Immunosuppressant, n (%)	36 (52.2)	75 (44.1)	1.273 (0.727-2.230)		0.398
HCQ, n (%)	30 (43.5)	85 (50.0)	0.769 (0.438-1.351)		0.361
aCL, n (%)	51 (73.9)	100 (58.8)	1.983 (1.069-3.680)		0.030
aβ2GPI, n (%)	46 (66.7)	57 (33.5)	1.009 (0.557-1.826)		0.977
LAC, n (%)	55 (79.7)	91 (53.5)	3.411 (1.763-6.596)		<0.001
Triple aPL positivity, n (%)	31 (44.9)	44 (25.9)	2.336 (1.301-4.195)		0.005
Platelet (×10^9^/L), median (IQR)	114.9 (63.0-172.0)	180.1 (104.3-237.5)	0.993 (0.989-0.996)		**<0.001**
Mean platelet volume (fl), median (IQR)	9.5 (8.2-10.9)	9.9 (9.0-10.9)	0.932 (0.784-1.108)		0.423
INR, median (IQR)	1.0 (0.9-1.1)	1.0 (0.9-1.2)	0.912 (0.418-1.988)		0.817
D-Dimer (ng/ml), median (IQR),	178.0 (87.0-543.0)	279.0 (96.0-557.5)	1.000 (1.000-1.000)		0.207
ESR increased, n (%)	24 (34.8)	74 (43.5)	0.692 (0.387-1.237)		0.214
CRP increased, n (%)	22 (31.9)	50 (29.4)	1.123 (0.614-2.056)		0.706
Low C3, n (%)	35 (50.7)	64 (37.6)	1.705 (0.969-2.999)		0.064
Low C4, n (%)	33 (47.8)	62 (36.5)	1.597 (0.906-2.813)		0.105
ANA positive, n (%)	43 (62.3)	103 (60.6)	1.076 (0.605-1.914)		0.804

IS, ischemic stroke; BMI, body mass index; IQR, interquartile range; aGAPSS, adjusted Global Anti-Phospholipid Syndrome Score; COPD, chronic obstructive pulmonary disease; HCQ, Hydroxychloroquine; LAC, lupus anticoagulant; aCL, anti-cardiolipin antibody; aβ2GPI, anti-β2-glycoprotein I antibody; aPL, antiphospholipid antibody; INR, international normalized ratio; ESR, erythrocyte sedimentation rate; CRP, C-reactive protein; C3, complement 3; C4, complement 4; ANA, antinuclear antibody.The provided bold values mean P-value < 0.05.

**Figure 2:**
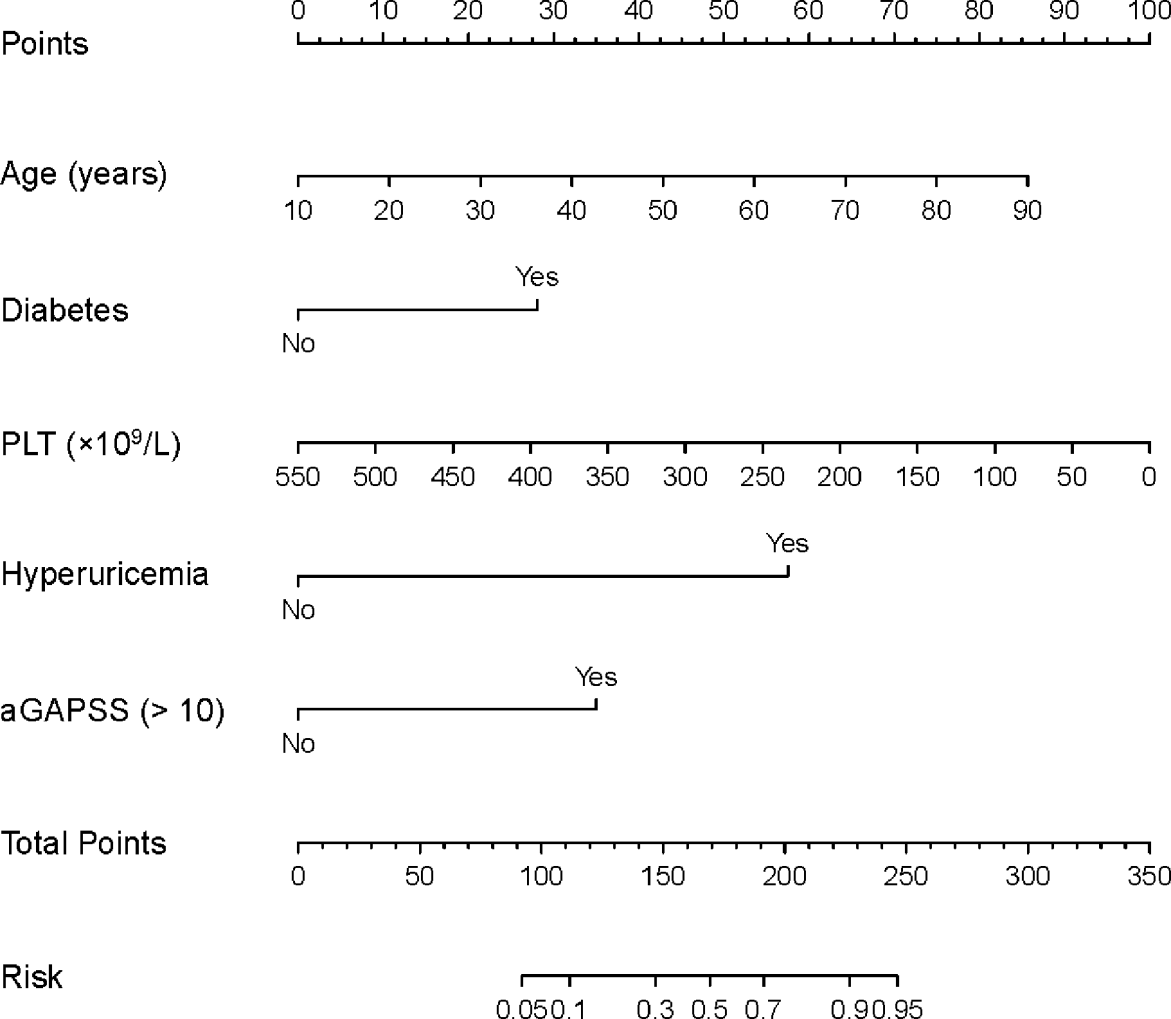
The nomogram for predicting the risk of IS in the training cohort. For each patient, we added up the points identified on the points scale for the five risk factors. Then, the risk probability of IS was obtained according to the “Total Points” axis of the nomogram. APS, antiphospholipid syndrome; IS, ischemic stroke; aGAPSS, adjusted Global Anti-Phospholipid Syndrome Score; PLT, platelet count.

In the univariate analysis, IS was associated with age (*P* < 0.001), sex (*P* = 0.017), diabetes (*P* < 0.001), hyperuricemia (*P* < 0.001), autoimmune disease (*P* = 0.020), the platelet count (*P* < 0.001), and the aGAPSS (*P* < 0.001; **Table 2**). For the training cohort, the AUROC for the ability of the aGAPSS to predict IS was 0.686 [95% confidence interval (CI), 0.623–0.744; *P* < 0.001]. The ROC curve analysis showed that the cut-off aGAPSS for IS prediction was 10, with a sensitivity of 75.4% and a specificity of 60%. In the multivariable regression analysis, independent predictors of IS in the training cohort were age (*P* < 0.001), diabetes (*P* = 0.030), hyperuricemia (*P* < 0.001), the platelet count (*P* = 0.001), and aGAPSS > 10 (*P* = 0.001; **Table 3**). All VIF values were below 1.26, indicating low degrees of collinearity among variables. We built a predictive nomogram for IS (the aGAPSS-IS) based on these five independent predictors (**Figure 2**). For each patient, we added up the points identified on the points scale for the five risk factors. Then, the risk probability of IS was obtained according to the “Total Points” axis of the nomogram.

**Table 3:** Multivariate analysis of IS occurrence based on the training cohort.

Variables	β Coefficient	Multivariate analysis	
		OR (95% CI)	P-value
Age (years)	0.041	1.042 (1.018-1.066)	**<0.001**
Gender	0.661	1.937 (0.889-4.217)	0.096
Diabetes	1.033	2.810 (1.102-7.160)	**0.030**
aGAPSS (> 10)	1.281	3.601 (1.677-7.731)	**0.001**
Hyperuricemia	2.150	8.584 (2.758-26.723)	**< 0.001**
Platelet counts (×10^9^/L)	-0.007	0.993 (0.988-0.997)	**0.001**
Autoimmune disease	0.322	1.380 (0.661-2.883)	0.391

IS, ischemic stroke; CI, confidence interval; OR, odds ratio; aGAPSS, adjusted Global Anti-Phospholipid Syndrome Score.The provided bold values mean P-value < 0.05.

### Validation of aGAPSS-IS score

**Figure 3:**
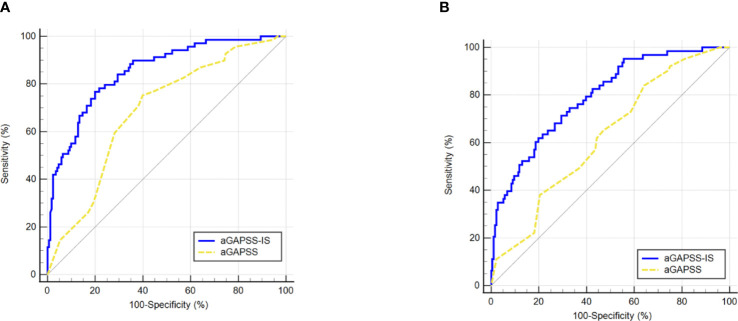
Comparison of the area under the receiver operating characteristic curve values between aGAPSS-IS score and aGAPSS in the training and the validation cohort. **(A)** In the training cohort, the aGAPSS-IS score had a larger AUROC than the aGAPSS [0.853 (95% CI, 0.802-0.896) *vs.* 0.686 (95% CI, 0.623-0.744), P < 0.001]; **(B)** In the validation cohort, the AUROC of aGAPSS-IS score was larger than the aGAPSS [0.793 (95% CI, 0.737-0.843) *vs.* 0.624 (95% CI, 0.560-0.656), P < 0.001]. aGAPSS, adjusted Global Anti-Phospholipid Syndrome Score.

**Figure 4:**
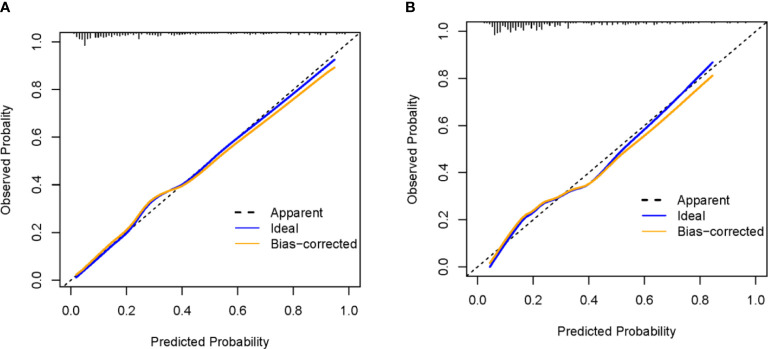
The calibration curve of the aGAPSS-IS score in the training and the validation cohort. **(A)** mean absolute error = 0.015 (training cohort); **(B)** mean absolute error = 0.028 (validation cohort).

**Figure 5:**
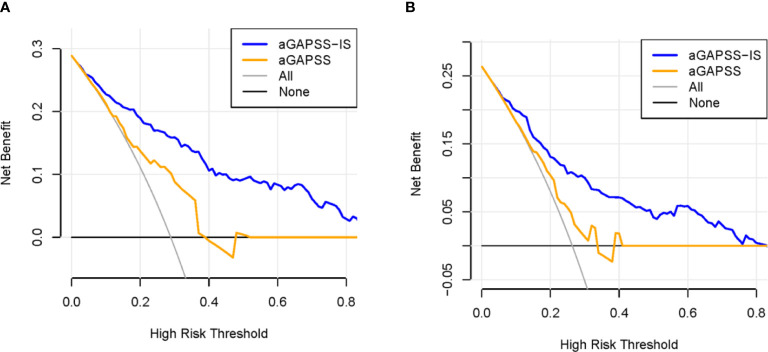
Comparison of the decision curves between the aGAPSS-IS score and aGAPSS in the training and the validation cohort. **(A)** For a threshold probability > 2%, application of the aGAPSS-IS score would add more net benefit to patients compared to the use of the aGAPSS in the training cohort. **(B)** For a threshold probability > 4%, the aGAPSS-IS score would provide more net benefit to APS patients than the aGAPSS in the validation cohort. aGAPSS adjusted Global Anti-Phospholipid Syndrome Score.

For the training and validation cohorts, the AUROCs for the aGAPSS-IS were larger than those for the aGAPSS [0.853 (95% CI, 0.802–0.896) *vs.* 0.686 (95% CI, 0.623–0.744) and 0.793 (95% CI, 0.737–0.843) *vs.* 0.624 (95% CI, 0.560–0.656), respectively, both *P* < 0.001], meaning that the aGAPSS-IS showed better discriminative capacity (**Figures 3A, B**). The calibration plot for the training cohort showed optimal agreement between the aGAPSS-IS–predicted probability and the observed probability of IS; the mean absolute error was 0.015 (**Figure 4A**). The plot for the validation cohort also showed excellent concordance between these probabilities, with a mean absolute error of 0.028 (**Figure 4B**). For the training cohort, the DCA demonstrated that the aGAPSS-IS provided more net benefits than the aGAPSS for IS prediction when the threshold probability was >2% (**Figure 5A**). Similarly, the aGAPSS-IS always had marked net benefits over the aGAPSS for IS prediction when the threshold probability was >4% (**Figure 5B**).

## Discussion

In the present study, we developed the clinical nomogram aGAPSS-IS for IS risk stratification for patients with APS. This nomogram was based on easily accessible data, including patient age, diabetes, hyperuricemia, platelet count, and aGAPSS. It showed better performance than the aGAPSS for IS prediction in an APS cohort. It could facilitate rheumatologists in making individualized decisions about the clinical management of patients with APS (e.g., whether neuroimaging is indicated during hospitalization) by weighing the probability of IS occurrence.

IS represents the most common and disabling arterial involvement in APS (28). It is vital to identify APS patients at high risk of stroke and adopt timely prophylactic treatment measures. The aGAPSS, based on conventional cardiovascular risk factors and aPL profile, was a widely accepted risk stratification score (10, 16, 29). Consistent with this, our results demonstrated that aGAPSS > 10 could predict IS in APS patients with a sensitivity of 75.4% and a specificity of 60%. However, the performance of aGAPSS in discriminating IS was dissatisfactory, perhaps due to the lack of consideration of some critical risk factors.

Age is a robust non-modifiable risk factor for IS in the general population; the risk of IS doubles every 10 years after the age of 55, and almost 75% of strokes occur in people aged > 65 years (30). Patients with diabetes are more prone to atherosclerosis and microangiopathy than are healthy people, and these vascular diseases deteriorate rapidly, leading to cardiovascular accidents. The prevalence of diabetes among patients experiencing IS is estimated to be 33% and was found to be associated closely with poor outcomes and stroke recurrence in this population (31). Thus, aging and diabetes contribute to the incidence of IS in patients with APS. Hyperuricemia has a dose-response relationship to cardiovascular disease (23) and is an accepted risk factor for venous thromboembolism (32, 33). According to a longitudinal study including 15773 participants, a 59.5-μmol/l increase in uric acid was associated with a 28% increase in total and cardiovascular mortality during ten years of follow-up (34). Monosodium urate crystals can induce neutrophil extracellular trap release (35), a very important mechanism for thrombosis in APS (36). Hence, hyperuricemia is another independent risk factor for IS in patients with APS. Thrombocytopenia is related significantly to IS and is a risk factor for thrombosis in patients with APS (37) and aPL carriers (12). A prospective study including 228 APS patients demonstrated that patients with thrombocytopenia had a higher risk of thrombotic events than those without [HR = 2.93, (95%CI:1.31-6.56)] (38). Phospholipids are integral parts of the platelet membrane, and the binding of aPL leads to the destruction of platelets and the release of microparticles, which play a procoagulant role in APS-related thrombotic events (39, 40). In our study, it was also an essential risk factor for arterial complications in the central nervous system.

The nomogram makes clinicians realize the critical role of traditional cardiovascular disease (CVD) risk factors for IS. CVD, especially stroke and coronary artery disease, is a leading cause of morbidity and mortality in APS (6). In the recent European League Against Rheumatism recommendations for managing CVD, the screening and strict control of traditional cardiovascular risk were highlighted in APS (41). However, CVD factors’ (e.g., hypertension and dyslipidemia) target achievement was suboptimal in APS, especially in high/very high-risk patients (42). Therefore, modifiable risk factors for arterial events, such as hypertension, diabetes, and hyperuricemia, should be strictly monitored and controlled in patients with APS to reduce the risk of IS.

This study has several limitations. First, it was retrospective rather than prospective, which may have attenuated the significance of the findings. Second, to make the nomogram convenient in clinical practice, we did not incorporate valuable data from other antibodies (e.g., anti-phosphatidylserine/prothrombin), which may lead to some information loss. Thirdly, in our study, only 53.9% (184/334) of thrombotic APS were under long-term anticoagulation. Similarly, in a prospective study of 1000 APS patients, 40.2% received oral anticoagulants during the first 5 years and 37.0% during the second 5 years of the follow-up period (6). Low rates of anticoagulation prescription in patients at high risk of thrombosis (e.g., APS, atrial fibrillation combined with IS) may exist in different regions (6, 43–45), posing a challenge for thrombosis prevention. The possible reasons may be lower levels of education, lower income, and prior antiplatelet use (43). There is no sufficient evidence that anticoagulation reduces the risk of IS in our study. Thus, anticoagulation was not included in the nomogram. More prospective studies are expected to explore the association between anticoagulation and IS incidence. Finally, this study was based on clinical information from a single center’s database and lacked external validation. Hence, we encourage the performance of multicenter studies to further validate the reliability and applicability of the aGAPSS-IS.

## Conclusion

The aGAPSS-IS may be a good tool for the identification of patients with APS at high risk of IS based on routinely available data. It may aid physicians in making individualized treatment decisions for patients with APS by weighing the probability of IS occurrence.

## Data availability statement

The raw data supporting the conclusions of this article will be made available by the authors, without undue reservation.

## Ethics statement

The studies involving human participants were reviewed and approved by ethics committee at Peking University People’s Hospital. The patients/participants provided their written informed consent to participate in this study.

## Author contributions

All authors were involved in the design of this study. CL and JZ conceived the original idea, supervised, and interpreted the result of this work. XS and YF performed the statistical analysis and wrote the manuscript. XS, YF, and JZ were involved in the assessment of ischemic stroke. YJ, GL, and ML contributed to clinical data collection pre-processing. YX gave advice in the statistical analysis and data interpretation. All authors contributed to the article and approved the submitted version.

## Funding

This work was supported in part by China International Medical Foundation (No. Z-2018-40-2101), National Natural Science Foundation of China (No.81871281), and Beijing Natural Science Foundation (7192211).

## Acknowledgments

The authors of this study would like to thank all the study participants.

## Conflict of interest

The authors declare that the research was conducted in the absence of any commercial or financial relationships that could be construed as a potential conflict of interest.

## Publisher’s note

All claims expressed in this article are solely those of the authors and do not necessarily represent those of their affiliated organizations, or those of the publisher, the editors and the reviewers. Any product that may be evaluated in this article, or claim that may be made by its manufacturer, is not guaranteed or endorsed by the publisher.
